# Liposomal Doxorubicin in the Treatment of Breast Cancer Patients: A Review

**DOI:** 10.1155/2013/456409

**Published:** 2013-03-26

**Authors:** Juan Lao, Julia Madani, Teresa Puértolas, María Álvarez, Alba Hernández, Roberto Pazo-Cid, Ángel Artal, Antonio Antón Torres

**Affiliations:** ^1^Medical Oncology Department, Miguel Servet University Hospital, Paseo Isabel la Católica, 1-3, 50009 Zaragoza, Spain; ^2^Aragón Institute of Health Sciences, Avda. San Juan Bosco, 13, planta 1, 50009 Zaragoza, Spain

## Abstract

Drug delivery systems can provide enhanced efficacy and/or reduced toxicity for anticancer agents. Liposome drug delivery systems are able to modify the pharmacokinetics and biodistribution of cytostatic agents, increasing the concentration of the drug released to neoplastic tissue and reducing the exposure of normal tissue. Anthracyclines are a key drug in the treatment of both metastatic and early breast cancer, but one of their major limitations is cardiotoxicity. One of the strategies designed to minimize this side effect is liposome encapsulation. Liposomal anthracyclines have achieved highly efficient drug encapsulation and they have proven to be effective and with reduced cardiotoxicity, as a single agent or in combination with other drugs for the treatment of either anthracyclines-treated or naïve metastatic breast cancer patients. Of particular interest is the use of the combination of liposomal anthracyclines and trastuzumab in patients with HER2-overexpressing breast cancer. In this paper, we discuss the different studies on liposomal doxorubicin in metastatic and early breast cancer therapy.

## 1. Background 

In the past years, we have seen significant advances in the understanding of neoplastic diseases and how they have been translated into improvements of therapy. An increasing number of more specific therapeutic options to manage different tumour types are now available, but classical chemotherapy (which is based on the administration of drugs that interfere with the cell's cycle, prevent its division, and eventually destroy them) remains, in general, a backbone option for many tumours. Chemotherapy side effects must not, however, be underestimated because its mechanism of action affects both tumour and normal cells as well. That is the reason why efforts to improve chemotherapy treatments have focused on designing drugs that are more specific against cancer cells to minimize toxic side effects. 

Liposomes were conceived as drug delivery systems to modify drug pharmacokinetics and distribution with the aim of reducing chemotherapy's toxicity. These liposomes improve the pharmacological properties of some cytostatic agents, allowing an increased proportion of the drug that may be delivered within the tumour tissue whilst substantially reducing the exposure of normal tissues.

Liposomes as a vehicle for delivering cytostatic agents were first described in the 1960s. They were initially used as carriers for lipophilic cytostatic agents, but their suitability for both hydrophilic and hydrophobic drugs was soon assessed. Liposomes can be either a membrane-based closed structure able to incorporate lipophilic drugs or may be built from the direct encapsulation of hydrophilic compounds within the internal aqueous compartment of vesicles [1–3]. 

Phospholipids are the major component of liposomes, which make them to be less toxic, biodegradable, and biocompatible. The bilayer of phospholipids prevents also the active form of the drug from breaking down before it reaches the tumour tissue and in this way exposure of the normal tissue to the drug is minimized. The therapeutic index of the drug is then increased by two mechanisms: on one hand, a greater amount of the active drug reaches the tumour cells and an increased cytotoxic effect is obtained and, on the other hand, side effects are also reduced as a consequence of the drug encapsulation. Liposomal formulations have an additional effect on drug metabolism by decreasing its enzymatic degradation [4]. 

Liposomes can be produced by different methods. Stability of both the bilayer and the incorporated drugs depends on lipid composition and cholesterol content. Their size ranges from 25 to 100 nM and is determined by the maximum quantity of drug stored within the membrane and its flexibility. The lower size limit avoiding liposomes may enter the normal capillary vessels whereas the upper limit is still within the tumour vasculature and enables the cytotoxic agent to reach the tumour bed; in order to produce its effect, the active drug needs to readily extravasate through the vascular defects present in the vessels surrounding cancer cells as a consequence of neoangiogenesis phenomena induced by neoplastic cells [5]. In this way, liposomes below this threshold have the potential to accumulate in the tumour bed after passive drug entry and boosted by impaired lymphatic drainage. This phenomenon has been described as “enhanced permeability plus retention effect” [6]. One more factor related to liposome's size is that the bigger they are the greater the uptake by the reticuloendothelial system and, therefore, more rapid the drug is metabolized [7]. 

As the time liposomes are retained in the circulatory system is reduced, the drug they are carrying might not reach cytotoxic levels in the tumour tissue. The size of the nanotransporter could be reduced, but then less drug quantity should be transported. One method that has proven to be effective in overcoming this obstacle without compromising the quantity of chemotherapeutic agent delivered to the tumour consists in coating these delivery systems with polymers, in particular, with polyethylene glycol (PEG) which allows liposomes to escape from the immune system and, therefore, increase “in vivo” circulating time [8]. Studies have shown that, when manufactured in this way, pegylated liposomes have a longer half-life than nonpegylated (ranging from a few hours to 45 hours) [9]. However, the presence of PEG may act as a barrier between the drug and the tumour cells hindering the delivery of the cytostatic. Therefore, future improvements should be directed to improve this aspect, particularly in the case of breast cancer. 

In this cancer, new liposomal formulations have been developed to facilitate the supply of the confined cytostatic agent using thermosensitive molecules. These formulations have proven to be effective in this tumour and their design keep them stable at normal body temperature of 37°C, but they become unstable at slightly higher temperatures as those existing inside the tumours. This system has also demonstrated a higher accumulation of the drug within the tumour and a facilitated release of the encapsulated drug [10]. 

An alternative strategy used to increase the therapeutic index of liposome-based drugs is based on improving the colocalization between the chemotherapeutic agent and the breast cancer cell. In some cases, this strategy can also include an improvement of the internalization of the drug into them as when cell surface receptors involved in endocytosis take part. 

In general, these formulations involve modifications of the liposome surface to contain ligands that are specifically recognized by receptors overexpressed in the breast cancer cell surface. Several of these strategies have been recently published. For example, anti-HER2 immunoliposomes have proven much more effective against HER2-overexpressing breast cancer cells when compared with nontargeted liposomes. In one study, targeted liposomes were formulated with a Fab of recombinant humanized anti-HER2 monoclonal antibody [11]. 

Estrogen receptor is a particularly attractive target as it is overexpressed in a large amount of breast cancer cell lines [12]. Several studies incorporating either estradiol or estrone to liposomes to use them as a ligand against estrogen-expressing breast cancer have been reported. In one study, the accumulation of these estrogen-targeted liposomes was approximately six times higher than that observed with nontargeted liposomes [13]. 

## 2. Metastatic Breast Cancer Treatment and Liposomal Anthracyclines Pharmacology

Breast cancer is a heterogeneous disease that includes a variety of biological types with different treatment options and clinical outcomes. Metastatic breast cancer (MBC) is a chronic and incurable disease, with a median survival of approximately 2-3 years. Although advances have been made in the management of MBC, long-term survivors are rare, with 5-year survival rates varying from 5% to 10%.

At present, prognosis and treatment selection are based on tumor biology and molecular characterization. In particular, multigene array and expression analyses have provided a molecular classification for breast tumor. The most important subtypes are luminal A and B, Her2/neu, and basal like [14, 15]. 

Characterization of tumor biology (estrogen and progesterone receptors, Ki-67 and Her2) and clinical history (past treatment, patient symptoms, and functional status) is critical for selecting treatment in MBC. Quality of life is an important issue to consider when choosing a therapeutic option. 

The targeted therapies, such as hormonal treatment of patients with hormone-sensitive tumors and trastuzumab in case of Her2 overexpression, represent a treatment of choice for a subset of selected patients. Nevertheless, cytotoxic chemotherapy remains the only therapeutic option in patients with triple negative condition or in those who progress after hormonotherapy. Anthracyclines and taxanes are the most active drugs for the treatment of MBC. For many decades, conventional anthracyclines, doxorubicin, and epirubicin have been an important mainstay in the treatment of breast cancer. They have proven to be effective for both metastatic and early disease, but their use has been limited because of the intrinsic cardiotoxicity [16]. 

Many strategies have been designed to curtail this effect. Encapsulating anthracyclines into liposomes, which allowed patients to receive much higher doses of an anthracycline delivered mainly into the tumour tissue with fewer side effects, has been one of these. Several formulations of liposome-encapsulated doxorubicin are available for its use in the clinical practice [17] which differ in pharmacological characteristics. 

Pegylated liposomal doxorubicin (PLD) (Caelyx) is doxorubicin hydrochloride encapsulated in liposomes with surface-bound methoxypolyethyleneglycol (MPEG). Doxorubicin hydrochloride is a cytotoxic anthracycline antibiotic derived from *Streptomyces peucetius* var. *caesius*. Pegylation avoiding liposomes may be detected by the mononuclear phagocyte system and thereby the blood circulating time is increased. Mean half-life of pegylated liposomes in humans is 55 hours. Its pharmacokinetic characteristics facilitate tissue accumulation and this has been demonstrated in tumour biopsies of Kaposi's sarcoma (KS) and bone metastases from breast cancer [18, 19]. 

Plasmatic pharmacokinetics of PLD in humans significantly differ from the original doxorubicin. Caelyx has a linear pharmacokinetic profile at lower doses (10–20 mg/m^2^) while in the dose interval of 20–60 mg/m^2^ PLD is nonlinear. Standard doxorubicin hydrochloride displays extensive tissue distribution (volume of distribution, 700–1.100 L/m^2^) and rapid clearance (24–73 L/h/m^2^). On the contrary, the distribution volume of PLD is limited mainly to the vascular fluid, and the elimination of doxorubicin from the blood depends on the liposomal carrier; doxorubicin becomes available for catabolism once the liposomes are extravasated and entered into the tissular compartment. 

At equivalent doses, plasma concentration and AUC values of PLD are significantly higher than those achieved with doxorubicin preparations. The pharmacokinetic profile of PLD determined in 18 patients with breast cancer (which was similar to a group of 120 patients with several tumour types) showed a mean half-life of 71.5 hours (range 45.2–98.5 hours). 

As already has been mentioned, the pegylated liposomal doxorubicin hydrochloride formulation allows the liposomes to circulate in the blood for extended periods of time. These pegylated liposomes are small enough (mean diameter of approximately 100 nM) to pass intact through the defective blood vessels supplying tumours. The entry of pegylated liposomes from blood vessels and their accumulation in tumours have been tested in mice bearing C-26 colon carcinoma tumours and in transgenic mice with KS-like lesions. The pegylated liposomes also combine a low permeability lipid matrix with an internal aqueous buffer system that keeps doxorubicin hydrochloride encapsulated as long as liposomes remain in the blood stream. 

Myocet (liposome-encapsulated doxorubicin citrate) is another form of encapsulated doxorubicin hydrochloride consisting of a drug delivery system with a highly rigid bilayer [20]. Myocet (LD) also provides a more prolonged circulating time than conventional doxorubicin and, in addition, liposome-encapsulation significantly modifies the biodistribution of doxorubicin, resulting in reduced toxicity. The clearance of LD was 5.1 ± 4.8 L/h and steady-state volume of distribution (*V*_*d*_) was 56.6 ± 61.5 L whereas, after conventional doxorubicin elimination and (*V*_*d*_) were 46.7 ± 9.6 L/h and 1.451 ± 258 L, respectively [21].

In animals (Table 1), liposome-encapsulated doxorubicin reduced the distribution to the heart and the gastrointestinal mucosa compared to conventional doxorubicin, while antitumor efficacy was maintained. However, when compared with conventional doxorubicin, LD did not prove to be more active in doxorubicin-resistant cell lines. 

Doxorubicin plasma pharmacokinetics in patients receiving LD showed a high degree of interpatient variability. Nonetheless, as a rule, total doxorubicin plasma levels were significantly higher with LD than with conventional doxorubicin, while free doxorubicin peak plasma levels were lower. Similarly, the peak levels of the main circulating doxorubicin metabolite, doxorubicinol (synthesized via aldo-keto-reductase) appeared in plasma later with LD than with conventional doxorubicin. Available pharmacokinetic data preclude settling strong conclusions regarding the relationship between plasma levels of total/free doxorubicin and its influence on the efficacy/safety of LD. 

## 3. Anthracycline Toxicity

Anthracyclines have a well-known toxicity profile. Their more frequent side effects include myelosuppression, mucositis, alopecia, and emesis. Other less frequent although highly relevant side effects are cardiotoxicity and the occurrence of secondary leukemias. 

The emetogenic potential of anthracyclines is moderate even though it is potentiated by other agents when administered in combination. The lowest blood cell count (nadir) is reached between 10 and 14 days after administration. Doxorubicin is a potent vesicant agent and its extravasation may cause necrosis of the skin and soft tissue. 

Anthracycline-induced cardiotoxicity was described for the first time in the 1970s [22]. Cardiac side effects can be divided into acute and late-onset events. Acute toxicity encompasses phenomena that are usually reversible and nonfatal, such as hypotension, tachycardia, and arrhythmias. The occurrence of symptoms of myocarditis (with or without accompanying pericarditis) in the immediate posttreatment days is less frequent but can lead to heart failure that is usually reversible. 

However, late-onset cardiotoxicity is the most relevant problem. It results in dilated cardiomyopathy that causes lethal congestive heart failure (CHF) in 75% of cases in the following 5 years and whose end-stage treatment may require a heart transplant [23]. This type of heart disease responds to a dosing and regimen-dependent pattern [22]. Toxicity is higher when anthracyclines are administered in bolus compared to regimens giving it as a continuous infusion and this seems to be related to the higher dose peak reached when administered in a short period of time. 

A number of factors that predispose to this toxicity have been identified. Specifically, they are hypertension, age below 15 or over 70 years, a history of radiotherapy to the mediastinum, and the concomitant use with other drugs such as cyclophosphamide, paclitaxel, or trastuzumab. In particular, when given with paclitaxel the risk of cardiotoxicity is higher when doxorubicin is administered just after paclitaxel instead of the opposite sequence. 

The earlier studies only recognized clinical-evident cardiac toxicity. 3-4% of patients treated with cumulative doses of 450 mg/m^2^ and up to 18% of those who received 700 mg/m^2^ presented with clinical heart failure [24]. The incidence of heart failure is lesser when epirubicin was used but occurred in a 0.7% of patients when cumulative doses of 660 mg/m^2^ were reached [25]. 

Anthracyclines cause some pathological changes prior to the occurrence of clinical cardiomyopathy that can be detected by different techniques: myocardial biopsy (Billingham scale); isotope ventriculography (MUGA scan) and echocardiography. Billingham published in 1978 a histological classification based on the findings observed in myocardial biopsies. Biopsy findings correlated fairly well with the cumulative doses of anthracyclines and were able to detect early damage to the myocardial cells. Early histological changes secondary to anthracyclines include cytoplasmic vacuolization and loss of muscle fibres from myocytes due to dilated sarcoplasmic reticulum. In more advanced stages, changes occur in cellular remodelling leading to left ventricular failure [26]. Such an invasive method has had no widespread use in daily clinical practice.

Isotope ventriculography (MUGA scan) has proven to be an easily reproducible and accurate technique in detecting anthracycline-induced cardiotoxicity [27]. Echocardiography is another noninvasive test used in the study and followup of anthracycline-induced cardiotoxicity. It is less accurate than ventriculography in the early detection of systolic dysfunction but allows assessing diastolic function whose decline seems to be a good predictor of early cardiac toxicity [28]. Other techniques such as antimyosin antibody scintigraphy or biomarkers such as troponin have been unable to predict early cardiotoxicity. 

The majority of recent studies accept as cardiotoxicity criteria a >20% reduction in the left ventricular ejection fraction (LVEF) as long as it remains above 50%, a >10% reduction if the resulting figure is below 50%, or when symptoms of CHF (congestive heart failure) occur [29]. Using these criteria, Swain calculated a 7.9% incidence of anthracycline-induced cardiotoxicity with a cumulative dose of 450 mg/m^2^; 15.7% with 500 mg/m^2^; 26% with 550 mg/m^2^, and 48% with 700 mg/m^2^ [30]. Shapiro et al. described cardiac toxicity incidence of 20% when the cumulative dose of doxorubicin in combination with cyclophosphamide reached 500 mg/m^2^ [31]. Adjuvant chemotherapy studies in which cumulative doses of doxorubicin did not exceed 300 mg/m^2^ showed an incidence of cardiomyopathy ranging from 0.2 to 0.9% [32]. Currently, cumulative doses that do not exceed 450–500 mg/m^2^ of doxorubicin or 900–1000 mg/m^2^ of epirubicin are accepted to be safe [25]. 

The simultaneous administration of other drugs potentiates anthracycline toxicity. The combined use of doxorubicin and paclitaxel was related to a rate of cardiotoxicity higher than predicted despite relatively low cumulative doses of doxorubicin [38]. This increased toxicity appeared to be caused by a pharmacokinetic interference between paclitaxel and doxorubicin resulting in higher doxorubicin and doxorubicinol plasma concentrations [39]. 

The combination of anthracyclines and trastuzumab has also been correlated with a higher rate of cardiotoxicity. In the pivotal study that compared doxorubicin and cyclophosphamide with or without trastuzumab in patients with overexpression of HER-2, a 23% rate of cardiac toxicity was observed with the combination compared with 7% in the arm not receiving trastuzumab [40]. Another study of the combination of trastuzumab with epirubicin and cyclophosphamide found that the combination with epirubicin 90 mg/m^2^ translated into 5% cardiac toxicity compared with only 1.7% when epirubicin was administered at 60 mg/m^2^ [41].

## 4. Liposomal Anthracyclines and Metastatic Breast Cancer

In patients with MBC, liposomal anthracyclines have shown similar efficacy and less toxicity when compared with conventional anthracyclines. Currently, three formulations with liposomal anthracyclines are available:

- Myocet: formulated with conventional liposomes;
- DaunoXome: liposomes with prolonged circulation half-lives;
- Caelyx/Doxil: with pegylated liposomes.

According to their respective product labelling, liposomal doxorubicin (LD, Myocet) was approved for the treatment of metastatic breast cancer; pegylated liposomal doxorubicin (PLD, Caelyx) for the treatment of advanced platinum-resistant ovarian cancer, advanced breast carcinoma, AIDS-related Kaposi's sarcoma, and multiple myeloma. 

In June 2000, Caelyx/Doxil received marketing authorisation in the US and subsequently in Europe, based on the results of a pivotal, randomised, controlled, and Phase III trial, which compared the efficacy of PLD with topotecan in the treatment of advanced ovarian cancer following failure of a platinum-containing regimen [42].

In MBC, both liposomal formulations have proven to be effective as single agent or in combination with other drugs for the treatment of either anthracycline-treated (progression-free interval of >6–12 months) or naïve patients [43–46].

Table 2 summarizes the trials that directly compared liposomal anthracyclines with conventional anthracyclines, either as monotherapy or combination. We shall review both, efficacy and toxicity, emphasizing data related to cardiac toxicity. Two Phase III studies have been published [33, 34] in which efficacy and toxicity of liposomal anthracyclines have been directly compared to conventional doxorubicin. There were no statistically significant differences between both treatments with respect to efficacy in terms of response rate, progression-free survival (PFS), or overall survival (OS). 

O'Brien et al. [33] reported the results of a noninferiority Phase III study in which 509 patients (p) with metastatic breast cancer were randomized to receive PLD at a dose of 50 mg/m^2^ every 4 weeks (254p) or conventional doxorubicin 60 mg/m^2^ every 3 weeks (255p). The study met its objective of noninferiority with PFS being 6.9 versus 7.8 months, respectively (HR 1.00; 95% CI 0.82–1.22). OS was comparable: 21 and 22 months for PLD and doxorubicin, respectively (HR 0.94; 95% CI 0.74–1.19). The objective response rate was also similar for PLD (33%) and doxorubicin (38%). Remarkably, the risk of cardiotoxicity was significantly higher in the conventional doxorubicin group (HR 3.6; 95% CI 1.58–6.31): forty-eight patients (19.6%) treated with doxorubicin developed cardiac toxicity compared with only 10p among those receiving PLD (*P* < 0.001). There were no patients with clinical heart failure in the PLD arm, while 10 patients (4%) in the conventional doxorubicin arm developed clinical heart failure. The number of patients to treat with PLD to avoid a doxorubicin-related cardiac event was 7. Also significant is that 16% of patients in the PLD arm received treatment for more than 9 months compared with only 1% in the doxorubicin arm and this was not linked to an increase in cardiac toxicity with PLD. In contrast, hand-foot syndrome incidence was higher in the PLD group (48% versus 2%). 

Harris et al. [34] compared the efficacy and safety of LD (75 mg/m^2^ every 3 weeks) with conventional doxorubicin (75 mg/m^2^ every 3 weeks) in 224 patients with metastatic breast cancer. Of them, 17% had received prior adjuvant or neoadjuvant treatment with anthracyclines. Response rate was 26% in both arms. PFS was 3.8 months in the LD arm compared to 4.3 in the conventional doxorubicin arm (*P* = 0.59). OS was 16 months in the LD arm versus 20 months in the conventional doxorubicin arm (*P* = 0.09). Myocardial biopsies were planned for patients with a LVEF reduction of >10% with absolute values above 50% or for those who had a LVEF reduction of >6% if the resulting LVEF was lower than 50%. In addition to the standard criteria for identifying cardiotoxicity, the presence of a grade of 2.5 or greater on the Billingham scale was included. The rate of cardiac events was favourable to the liposomal anthracycline arm (13 versus 29%, *P* = 0.0001) with a clinical heart failure rate of 5.9 versus 15%. When the heart biopsies performed were analyzed, the proportion of patients with a value of 2.5 on the Billingham scale was 26 versus 71% (*P* = 0.02) favouring the liposomal formulation. The mean cumulative dose until toxicity occurred was calculated at 570 mg/m^2^ for doxorubicin and 785 mg/m^2^ for liposomal doxorubicin. 

Some other Phase III studies [35–37] compared efficacy and toxicity of liposomal anthracyclines in combination with other cytostatic agents (docetaxel or cyclophosphamide) with combinations with conventional anthracyclines or other drugs. Inclusion criteria for these studies were not identical, mainly regarding prior treatment allowed. Studies by Chan et al. and Batist et al. included patients not previously treated with anthracyclines; Sparano et al., however, randomized patients previously treated with anthracyclines during adjuvant or neoadjuvant therapy as long as progression-free interval was above 12 months. As Table 2 shows, we can see that overall efficacy of liposomal anthracyclines is similar to the efficacy of conventional formulations when combined with other cytostatic agents. Of note, in Chan's study PFS was even higher in the group treated with Myocet plus Cyclophosphamide. 

In Batist's study [35], 30% of patients presented any cardiotoxicity risk factor and 10% had received prior anthracyclines (adjuvant) with a mean cumulative dose of 240 mg/m^2^. Here, 21% of patients treated with conventional doxorubicin had some grade of cardiotoxicity compared to 6% in the group receiving liposomal doxorubicin (*P* = 0.0001). In the control arm, 3.2% of patients developed clinical heart failure compared with 0% in the liposomal doxorubicin arm. The analysis of patients with any cardiac risk factor showed an even greater difference between both drugs with a HR of 16.1. The mean cumulative dose calculated for 50% of patients presenting with cardiotoxicity was much higher in the group receiving liposomal doxorubicin (2.220 mg/m^2^ versus 480 mg/m^2^). 

Eventually, the same author published in 2006 [47] retrospective data from the analysis of 68 patients that had been included in the Phase III study and had been treated with adjuvant anthracyclines. Cardiac toxicity was lower in patients treated with liposomal doxorubicin (22 versus 39%, HR: 5.4, *P* = 0.001). Four patients developed congestive heart failure, 3 of them in the doxorubicin arm. The calculated mean cumulative dose until cardiotoxicity occurrence was 580 mg/m^2^ for doxorubicin and 780 mg/m^2^ for the liposomal formulation (HR: 4.8, *P* = 0.001). 

A further Phase III study [36] randomized 160 patients to receive cyclophosphamide 600 mg/m^2^ plus either epirubicin 75 mg/m^2^ or liposomal doxorubicin 75 mg/m^2^. No significant differences were observed in the rate of asymptomatic reduction in LVEF (11 versus 10%). In this study, no patient developed clinical heart failure. It must be noted that epirubicin dosing was lower than the equipotent doxorubicin. 

In 2010, the Cochrane Library reported a systematic review of the different anthracycline compounds and their cardiotoxicity [48]. Studies by Harris and Batist were analyzed together and authors concluded that nonpegylated liposomal anthracyclines reduced the overall risk of cardiotoxicity (RR = 0.38, *P* < 0.0001) and the risk of clinical heart failure (RR = 0.20, *P* = 0.02). 

Efficacy and safety of pegylated liposomal doxorubicin (PLD) combined with other cytostatic agents were studied in two Phase III studies. 

Sparano et al. [37] randomized 751 patients previously treated with anthracyclines (as adjuvant or neoadjuvant) with a PFI over 12 months to receive either docetaxel 75 mg/m^2^ (373p) or the combination of PLD 30 mg/m^2^ plus docetaxel 60 mg/m^2^ every 21 days (378p) until disease progression or unacceptable toxicity occurred. Combined treatment improved PFS significantly from 7.0 to 9.8 months (HR 0.65; 95% CI, 0.55 −0.77; *P* < 0.00001). OS was similar: 20.6 months in the docetaxel arm and 20.5 in the combined treatment arm (HR 1.02; 95% CI, 0.86–1.22). The incidence of hand-foot syndrome was higher in the combined treatment arm (24% versus 0%) and symptomatic cardiac toxicity was similar: 4% in the docetaxel group and 5% in the PLD-docetaxel group. 

Patients with metastatic breast cancer progressing after taxanes and anthracyclines had fewer treatment options and often anthracyclines were not used again, due to the cumulative risk of cardiotoxicity. Based on the safety and efficacy data for PLD, a Phase III study was proposed [49] in which 301 patients with metastatic breast cancer progressing to taxanes (<6 months) were randomized to receive one of the following three alternatives: PLD 50 mg/m^2^ every 4 weeks (150p); vinorelbine 30 mg/m^2^ every week (129p); or mitomycin-C 10 mg/m^2^, on days, on 1 and 28 plus vinblastine 5 mg/m^2^ on days 1, 14, 28, and 42 every 6–8 weeks (22p). 83% of patients had received prior anthracyclines, in 10% of them cumulative doses above 450 mg/m^2^ had been reached. No patient treated with PLD showed clinical symptoms of cardiotoxicity. PFS was similar (2.86 months in the PLD group versus 2.53 months in the other two control groups) (HR 1.26; 95% CI, 0.98–1.62). In the subgroup of patients not previously treated with anthracyclines (44p), PFS was higher in the PLD arm (5.8 months) compared with the control arms (2.1 months) (*P* = 0.01). OS was slightly higher with PLD (11 months) versus control arm (9 months), albeit not statistically significant (*P* = 0.93). The objective response rate was similar: 10% for PLD versus 12% for the control arm. 

More recently an Austrian observational study was published [50] in which 129 patients with metastatic breast cancer treated with PLD were analyzed. 70% presented 2 or more cardiovascular risk factors. Despite this, only 4% of patients had some degree of cardiotoxicity and only 2 cases of clinical heart failure were reported. 

Alba et al. [51], on behalf of GEICAM, published a Phase III study exploring the role of PLD as maintenance therapy. Eligible patients had previously received a sequential scheme based on 3 cycles of doxorubicin 75 mg/m^2^ followed by 3 more cycles of docetaxel 100 mg/m^2^. Patients, who had not progressed during this first part, were randomized to receive pegylated liposomal doxorubicin 40 mg/m^2^  × 6 cycles or nothing. TTP from randomization of the 155 p was 8.4 versus 5.1 months favouring the maintenance treatment arm (*P* = 0.0002). No differences in OS were found. Six patients had reduced LVEF ≥ 10%, 5 of them in the arm of PLD. In 2 of the patients treated with PLD, a LVEF reduction below 50% during treatment was found, although both recovered within 6 months. There was no clinical cardiac toxicity.

## 5. Liposomal Anthracyclines and Trastuzumab

In HER2-postive breast cancer, the addition of trastuzumab to chemotherapy significantly increases response rate, time to progression, and overall survival compared with chemotherapy alone. However, when trastuzumab is combined with anthracyclines there is an increased risk of cardiac toxicity. Slamon et al. [40] randomized 469p with metastatic breast cancer and HER2 overexpression to receive standard treatment (anthracyclines/cyclophosphamide or paclitaxel) with or without trastuzumab. The addition of trastuzumab increased PFS (7.4 months versus 4.6 months, *P* < 0.001) and OS (25.1 versus 20.3 months, *P* = 0.046), but with an increased rate of cardiotoxicity in the group receiving the anthracycline and trastuzumab combination (27%). These results limited the use of anthracyclines in HER2-positive breast cancer, and in consequence non-anthracycline-based regimens such as TCH [52, 53] were designed. As anthracyclines showed a high level of activity in this subgroup of patients, other strategies were developed also to design regimens using less cardiotoxic anthracyclines such as epirubicin (a less cardiotoxic analog than doxorubicin) at limited doses or liposomal anthracyclines in combination with trastuzumab [54] which will be further analyzed. 

Several studies with a small number of patients explored the viability of combination regimens with liposomal anthracyclines and trastuzumab in metastatic breast cancer. LD (Myocet) proved to be as effective as and less cardiotoxic than conventional anthracyclines when combined with trastuzumab in 4 Phase I/II studies. 

The first was a Phase I/II study by Theodoulou et al. [55] that included 37 patients with HER2-positive metastatic breast cancer, 14 patients had been previously treated with adjuvant doxorubicin (<240 mg/m^2^) and 17 patients with one or two lines of prior chemotherapy for advanced disease (11 with trastuzumab). Myocet 60 mg/m^2^ was administered every 3 weeks plus trastuzumab 2 mg/Kg weekly. Response rate was 58% (95% CI 41–75%). A LVEF reduction of >10% was observed in 10 patients (25%). Five patients (12%) presented with a LVEF < 50%, 4 of them had been pretreated with anthracyclines; 2 patients (5%) withdrew from the trial due to cardiac toxicity. 

Another Phase I/II trial [56] included 69 patients with locally advanced or metastatic disease who had received no prior treatment. The treatment regimen chosen for the Phase II was trastuzumab combined with liposomal doxorubicin 50 mg/m^2^ every 21 days and paclitaxel 80 mg/m^2^ weekly. Response rate was 98.1% (95% CI 90.1–99.9). Median time to progression was 22.1 months (95% CI 16.4–46.3) in metastatic patients and had not yet reached in locally advanced patients by the time of publication. No cases of treatment-related clinical heart failure were observed. Twelve patients presented with an asymptomatic reduced LVEF, 8 of them recovering up to values of 50% or greater within a mean of 9 weeks. 

Venturini et al. [57] conducted a Phase II study in 31 patients with first-line metastatic disease to evaluate the safety and efficacy of combining trastuzumab, LD, and docetaxel. Eight cycles of chemotherapy were administered, followed by trastuzumab monotherapy to complete 52 weeks of treatment. The response rate was 65.5% with a TTP of 13 months. Five of the 31 patients experienced a ≥ 20% reduction from baseline or an absolute LVEF < 45%.

Another Phase I-II trial with LD in combination with trastuzumab and docetaxel was conducted by Amadori et al. [58]. Forty-five patients with metastatic breast cancer received weekly trastuzumab associated with LD 50 mg/m^2^ every 3 weeks and docetaxel 30 mg/m^2^ on days 2 and 9. The response rate was 55.6% with a TTP of 10.9 months. Only 2 patients had a decrease in LVEF below 50%.

Similarly, the use of PLD combined with trastuzumab may reduce the incidence of cardiotoxicity while maintaining a similar efficacy. We shall describe a series of small Phase II studies that investigated this alternative. Chia et al. [59] included 30 patients with HER2-positive metastatic breast cancer (MBC), 13 of them previously treated with adjuvant anthracyclines (<300 mg/m^2^). PLD 50 mg/m^2^ was given every 4 weeks and trastuzumab 2 mg/Kg weekly for 6 cycles. Response rate was 52% and PFS 12 months. The most frequent toxicities were grade 3 hand-foot syndrome (30%) and grade 3/4 neutropenia (27%). Cardiac toxicity incidence was 10% and in no case was symptomatic. Andreopoulou et al. [60] included 12 patients with MBC on first- and second-line therapy, 7 treated with adjuvant anthracyclines and 7 with prior trastuzumab for metastatic disease. They received treatment with PLD every three weeks and trastuzumab weekly achieving 66% disease stabilization. 25% presented with grade 2 cardiac toxicity. Stickeler et al. [61] enrolled 16 patients with HER2-positive metastatic breast cancer; 5 had received prior chemotherapy for advanced disease (2 of them received anthracyclines <400 mg/m^2^). PLD 40 mg/m^2^ was administered every 4 weeks for 6–9 cycles plus trastuzumab weekly; response rate was 50%, PFS 9.67 months, and OS 16.23 months. Christodoulou et al. [62] studied trastuzumab combined with PLD administered at a dose of 30 mg/m^2^ every three weeks. All patients should have received first-line chemotherapy for advanced disease or have relapsed before the end of the year of taxane-based adjuvant treatment. The response rate was 22%, PFS 6.5 months, and OS 18.7 months. There were no episodes of LVEF reduction in any of the patients.

Wolff et al. [63] published a Phase II study (ECOG E3198) in which 84 patients with HER2-positive or negative MBC on first-line therapy were included and who had not been previously treated with anthracyclines. PLD was administered at a dose of 30 mg/m^2^ together with docetaxel 60 mg/m^2^ every three weeks (maximum of 8 cycles) plus trastuzumab (46p) or without it (38p) according to HER2 expression. Response rate was 47.4% in the arm without trastuzumab (95% CI 31.0–64.2%) and 45.7% in the arm with trastuzumab (95% CI 30.9–61%). PFS was 11 months (95% CI 8.6–12.8 months) and 10.6 months (95% CI 15.6-15.7), respectively. Median OS was 24.6 months (95% CI 14.7–37.3) and 31.8 months (95% CI: 23.7–44.9 months). There was only one case of heart failure who was a HER2-negative patient. The addition of trastuzumab in patients with HER2 overexpression was not associated with higher cardiac toxicity but was related to a higher incidence of hand-foot syndrome.

Recently, Martín et al. [64] published a Phase II study (GEICAM 2004/05) which included 48 patients in first-line metastatic disease. PLD was administered at doses of 50 mg/m^2^ in combination with cyclophosphamide 600 mg/m^2^ every 4 weeks along with weekly trastuzumab. The response rate was 68.8%, the TTP was 12 months and OS of 34.2 months. There were no symptomatic cardiac events. Eight patients (16.7%) had decreased LVEF grade 2; six of them had been previously treated with anthracyclines. Seven of the 8 patients recovered cardiac function. 

## 6. Early Breast Cancer

A number of small studies of neoadjuvant treatment with liposomal anthracyclines for locally advanced breast cancer have been published. The Phase I study by Possinger et al. [65] included 20 patients receiving a combination of LD 60 mg/m^2^ plus docetaxel 75 mg/m^2^ on day 1 and gemcitabine 350 mg/m^2^ on day 4, every 3 weeks. The use of colony-stimulating factors was mandatory. Response rate was 88%. No cardiotoxicity was observed, but there was significant haematological toxicity (29%) and stomatitis (28%). Another Phase II study published by Gogas et al. [66] included 35 patients receiving treatment with PLD 35 mg/m^2^ in combination with paclitaxel 175 mg/m^2^ every 3 weeks for 6 cycles. Response rate was 71%. Grade 3 toxicity was cutaneous (11%), hand-foot syndrome (9%), and leukopenia (11%). No cardiac toxicity was observed. 

## 7. HER-2-Positive Early Breast Cancer

There has been a greater interest in the use of liposomal anthracyclines in early breast cancer overexpressing HER2 oncogene, as this subgroup of patients could obtain the greatest benefit from treatment with anthracyclines [67] and combining them with trastuzumab may be difficult due to the high cardiotoxicity that could be induced. 

Our group designed a Phase I-II study (GEICAM 2003-03) in patients with early breast cancer to be given as neoadjuvant therapy to deal with the dose variability of LD (Myocet) in combination with other drugs and the lack of evidence for a maximum tolerated dose when combined with docetaxel and trastuzumab [68, 69]. The results for Phase I after the inclusion of 19 patients with stages II and IIIA HER2-positive breast cancer determined the recommended dose for Phase II to be LD 50 mg/m^2^ plus docetaxel 60 mg/m^2^ every three weeks with standard dose trastuzumab when prophylactic pegylated-filgrastim was administered. Only one of the 19 patients presented with cardiac toxicity and it was an asymptomatic grade 2 reduction in LVEF. Pathologic complete response rate in the primary tumour and axillary lymph nodes was 33%. With such stimulating data on activity and safety, Phase II of the study was completed. Fifty-nine patients with HER2-positive breast cancer were included: stages II, 40p and IIIA, 19p. The recommended dose from prior Phase I was administered every 21 days: liposomal doxorubicin 50 mg/m^2^, docetaxel 60 mg/m^2^ and trastuzumab 2 mg/kg/weekly along with prophylactic pegylated-filgrastim. The clinical response rate was 86% and radiological response rate was 81%. No patient progressed during treatment. All patients underwent surgery which was conservative in 42 cases. Seventeen patients (29%, 95% CI 17.2–40.4) obtained a pathologic complete response in the breast tumour (G5 Miller and Payne) and 16 of them (27%, 95% CI 15.8–38.4) also obtained a pathologic complete response in the axillary lymph nodes. An additional 15% obtained a grade 4 Miller and Payne response in the primary tumour. Neutropenia was the most significant grade 3-4 haematological toxicity (17 patients, 29%), but only 3 developed neutropenic fever. Grade 3 nonhaematological toxicity was infrequent: asthenia in 5 patients, nausea in 3, diarrhoea in 3, and stomatitis in one patient. Grade 2 (>20% reduction of the baseline value or reduction below the normal value of 50%) asymptomatic reduction of LEVF was observed in 5 patients (9%) and treatment was withheld in only one of them. By the end of treatment, 3 of the patients had recovered a LVEF greater than 50%. There were no episodes of clinical heart failure. 

Finally, a Phase II randomized study published by Rayson et al. [70] provided us with information regarding cardiotoxicity of the combination of PLD plus trastuzumab used concomitantly in adjuvant therapy for intermediate-risk breast cancer with HER2 overexpression and either negative or positive lymph nodes. 181 patients with a baseline LVEF >55% were included. They were randomized (1 : 2) to arm A: doxorubicin 60 mg/m^2^ plus cyclophosphamide 600 mg/m^2^ every 21days, four cycles or arm B: PLD 35 mg/m^2^ plus cyclophosphamide 600 mg/m^2^ every 21 days, four cycles plus trastuzumab 2 mg/kg weekly for 12 weeks. Both groups subsequently received paclitaxel 80 mg/m^2^ plus trastuzumab for 12 additional weeks, followed by trastuzumab in monotherapy to complete one-year therapy. The main objective of the study was cardiac toxicity: comparing the rate of cardiac events and/or the percentage of patients who were unable to complete one-year treatment with trastuzumab. The incidence of cardiac toxicity was 18.6% with doxorubicin (95% CI 9.7%–30.9%) versus 4.2% with PLD (95% CI 1.4%–9.5%) (*P* = 0.0036). Among the 16 patients who had a cardiac event (11 in the conventional doxorubicin arm and 5 in the PLD arm), 8 were over 55 years old. All the events occurred after the 4th course of therapy. One of the events was a myocardial infarction with subsequent clinical heart failure (this occurred in arm B). Of the remaining 15 cases, 7 were recorded as >10% reduction from baseline LVEF with absolute values of <50% (3 of them developing clinical symptoms were classed as NHYA class II heart failure). The other 8 cases were classed as asymptomatic (NYHA class I). There were no cardiotoxicity-related deaths. The LVEF mean value was similar in both groups (64.0%, PLD + C + H/T + H and 64.4%, A + C/T + H). Mean reduction of LVEF values after the 8th cycle (end of chemotherapy) was significantly higher in patients receiving conventional doxorubicin (5.6% versus 2.1%; *P* = 0.0014). Cardiac safety analysis for this study suggested that administering trastuzumab concomitantly with PLD in the tested regimen was feasible, caused less cardiotoxicity in the short term, and avoided the premature interruption of treatment with trastuzumab when compared with a standard regimen such as A + C/T + H. The authors concluded that this strategy of incorporating early and concomitantly a liposomal anthracycline plus trastuzumab was safe, but its possible clinical role should be properly investigated in a randomized Phase III trial versus a nonanthracycline regimen such as TCH. 

## 8. Conclusions

Liposome-based drug delivery systems are able to modify the pharmacokinetics and pharmacodynamics of cytostatic agents, enabling us to increase the concentration of the drug released into the neoplastic tissue and, at the same time, reducing the exposure of normal tissue to the drug. 

Anthracyclines are important agents in the treatment of both metastatic and early breast cancer, but cardiotoxicity remains one of the major limitations for their use. Liposome encapsulation is one of the strategies designed to minimize this side effect. There are several liposome-encapsulated doxorubicin formulations available which show different pharmacological characteristics. The most commonly used are liposomal doxorubicin (Myocet) and pegylated liposomal doxorubicin (Caelyx). 

In patients with metastatic breast cancer, liposomal anthracyclines have proven to be as effective and less toxic when compared face to face with conventional anthracyclines, allowing a longer period of treatment and a higher cumulative dose of the anthracyclines. The combined analysis of available data indicates an overall reduction in risk for both cardiotoxicity (RR = 0.38, *P* < 0.0001) and clinical heart failure (RR = 0.20, *P* = 0.02). The safety of liposomal anthracyclines endorsed its use in patients with some cardiac risk factors. 

In HER2-positive breast cancer, the addition of trastuzumab to chemotherapy significantly increased response rate, progression-free survival, and overall survival. Initial studies demonstrated synergy when trastuzumab was combined with anthracyclines, but their excessive cardiac toxicity limited their use and nonanthracycline therapeutic strategies were designed. 

Liposomal anthracyclines have proven to be effective and safe when combined with trastuzumab both in advanced and early breast cancer. Of particular interest is the use of the combination of liposomal anthracyclines plus trastuzumab in patients with early and HER2-overexpressing breast cancer, as this is probably the subgroup that would benefit most from a treatment with anthracyclines. The potential clinical benefit of anthracyclines in this setting should be investigated in a clinical trial comparing a regimen with liposomal anthracyclines versus a nonanthracyclines combination. 

## Figures and Tables

**Table 1:** Comparison of AUC and *t*_1/2_ in various tissues in dogs following the administration of TLC D-99 and conventional doxorubicin. Single dose 1.5 mg/kg (30 mg · m^−2^), IV [18].

Tissues	TLC D-99		Doxorubicin		Ratio of AUC_0→*T*last_ (TLC D-99/Dox)
	AUC_0→*T*last_ (uM eq-h)	*T*_1/2_ (h)	AUC_0→*T*last_ (uM eq-h)	*T*_1/2_ (h)	
Liver	539	79	377	97	1.42
Spleen	5,087	92	559	52	9.07
Bone marrow	1,913	86	392	75	4.86
Lymph nodes	896	211*	653	196*	1.38
Myocardium (left ventricle)	208	59	313	50	0.66
Myocardium (right ventricle)	189	62	282	54	0.67

*Due to short sampling intervals relating to apparent *t*_1/2_, these values are estimated. TLC D-99: nonpegylated liposomal doxorubicin.

**Table 2:** Trials that directly compared liposomal anthracyclines with conventional anthracyclines, either in monotherapy or combination.

Author	Trial phase	Treatment regimen	Patients' characteristics	PFS	OS	RR	Toxicity
O'Brien et al. [33]	III	PLD (50 mg/m^2^/4w) versus ADR (60 mg/m^2^/3w)	Stage IV	6.9 m versus 7.8 m	21 m versus 22 m	33% versus 38%	Cardiac: 4.7 versus 19.6% CHF: 0% versus 4%

Harris et al. [34]	III	LD (75 mg/m^2^/3w) versus ADR (75 mg/m^2^/3w)	Stage IV (17% ADR previous)	3.8 m versus 4.3 m	16 m versus 20 m	26%	Cardiac: 13 versus 29% CHF: 5.9 versus 15% Billingham > 2.5: 26 versus 71%

Batist et al. [35]	III	LD (60 mg/m^2^) + CTX (600 mg/m^2^) versus ADR (60 mg/m^2^) + CTX (600 mg/m^2^)	Stage IV (10% ADR previous) (30% CRF)	5.1 m versus 5.5 m	19 m versus 16 m		Cardiac: 6 versus 21% (*P* < 0.05) CRF: 0 versus 3.2%

Chan et al. [36]	III	LD (75 mg/m^2^) + CTX (600 mg/m^2^) versus EPI (75 mg/m^2^) + CTX (600 mg/m^2^)	Stage IV (No ADR previous)	7.7 m versus 5.6 m	18.3 m versus 16 m	46 % versus 39 %	Cardiac: 11 versus 10% No CRF

Sparano et al. [37]	III	Docetaxel (75 mg/m^2^) versus Docetaxel (60 mg/m^2^) + PLD (30 mg/m^2^)	Stage IV (100% ADR previous)	7 m versus 9.8 m	20.6 m versus 20.5 m		Cardiac: 4 versus 5% PPS: 0 versus 24%

PLD: pegylated liposomal doxorubicin; LD: liposomal doxorubicin; ADR: adriamycin; EPI: epirubicin; CTX: cyclophosphamide; PFS: progression-free survival; OS: overall survival; RR: response rate; PPS: plantar-palmar syndrome; CHF: clinical heart failure; and CRF: cardiac risk factor.
